# Changes in the Distribution Range of the Genus *Cardiocrinum* in China Under Climate Change and Human Activities

**DOI:** 10.3390/biology14050581

**Published:** 2025-05-21

**Authors:** Yuxin Zhang, Shujian Zhang, Haiyan Xiao, Heng Li, Da Liao, Yuxi Xue, Xinyi Huang, Qitao Su, Yian Xiao

**Affiliations:** Key Laboratory of Jiangxi Province for Biological Invasion and Biosecurity, School of Life Sciences, Jinggangshan University, Ji’an 343009, China

**Keywords:** *Cardiocrinum*, MaxEnt moldel, habitat distribution, environment variables

## Abstract

*Cardiocrinum* are perennial herbaceous plants of the Liliaceae family with high ornamental, nutritional, and medicinal value. However, critical knowledge gaps remain regarding the following: (1) the fine-scale habitat preferences of *Cardiocrinum*; (2) the key ecological drivers influencing their growth and distribution. The MaxEnt software 3.4.1 was used to simulate the current and future suitable habitats of *Cardiocrinum*, evaluate the impacts of environmental changes on its distribution, and determine the distribution changes under climate change scenarios. The AUC value of the model used in the current study was >0.98, which indicates that the model had good accuracy. The results show that as a typical understory herb, precipitation in the warmest quarter (bio18) and temperature seasonality (bio04) are the main factors affecting the distribution of *Cardiocrinum*. In addition, *Cardiocrinum giganteum* and *Cardiocrinum giganteum* var. *yunnanense* are also affected by slope and human activity. Under the SSP126, SSP245, and SSP585 climate scenarios, the suitable habitat areas of *Cardiocrinum cathayanum* and *C. giganteum* showed an increasing trend. The suitable habitat area of *C. giganteum* var. *yunnanense* increased under the SSP126 climate scenario; however, it substantially declined in SSP245 and SSP585 scenarios. The distribution area of *Cardiocrinum* shifted to higher latitudes. The centroid of *C. cathayanum* shifted more than 5 degrees of latitude during SSP585 2081s, while the centroid of *C. giganteum* and *C. giganteum* var. *yunnanense* did not shift more than 2 degrees of latitude. In addition, the centroid longitudes of *C. giganteum* and *C. giganteum* var. *yunnanense* shifted westward under the three climate scenarios. There is ecological niche differentiation among *C. cathayanum* and others, whilst *C. giganteum* and *C. giganteum* var. *yunnanense* have overlapping ecological niches. In the future, we will strengthen the protection of wild *Cardiocrinum* resources in accordance with environmental factors and suitable habitats for *Cardiocrinum*.

## Simple Summary

The genus *Cardiocrinum* comprises three recognized species, with their native distributions primarily concentrated in eastern and southwestern China’s forest ecosystems. In this study, the Maxent model was used to study the distribution and suitable area change in *Cardiocrinum* under climate change. The study findings showed that precipitation, temperature, and human activities significantly impacted the *Cardiocrinum* distribution. In addition to the relative reduction in the suitable habitat area of *Cardiocrinum giganteum* var. *yunnanense*, the suitable habitat area of *Cardiocrinum cathayanum* and *Cardiocrinum giganteum* will increase significantly and in the 2081–2100 SSP585 climate, and the area ideal for *Cardiocrinum cathayanum* habitat will increase greatly. Compared with the current period, this indicates that *Cardiocrinum* will undergo significant range shifts, with suitable habitats migrating toward higher altitude in the future. The niche consistency test of *Cardiocrinum* showed that there was niche differentiation between *Cardiocrinum cathayanum* and the other two, and there was no niche differentiation between *Cardiocrinum giganteum* and *Cardiocrinum giganteum* var. *yunnanense*.

## 1. Introduction

*Cardiocrinum* are perennial herbaceous plants of the family Liliaceae, which are known as the “Prince of Lilies” due to their striking size, beautiful white flowers, and aromatic smell [1]. According to the Flora of China, there are two species of *Cardiocrinum* in China, namely *Cardiocrinum giganteum* and *Cardiocrinum cathayanum*. *C. giganteum* has a variant of *Cardiocrinum giganteum* var. *yunnanense* [2]. *C. giganteum* var. *yunnanense* is a near-threatened plant on the Informatin System of Chinese Rare and Endangered Plant (ISCREP) [3], and *C. cathayanum* is classified as a second-class nationally protected wild plant in China [4]. The plants of *Cardiocrinum* are perennial herbaceous plants in the forest. They like humid, cold, and shaded environments. They often grow under deciduous broad-leaved forests or beside streams on the edge of forests [1]. *Cardiocrinum* have esthetic, edible, and medicinal value. Their bulbs and tender leaves are rich in nutrients such as protein, amino acids, lily glycosides, starch, vitamin C, and vitamin B, which can be eaten as vegetables [5]. The biflavonoid compounds (CGY-1 and CGY-2) extracted from the bulbs of *Cardiocrinum* have good antitussive effects [6]. Field surveys have found that due to the expansion of human activities and the excavation and utilization of wild *Cardiocrinum*, wild *Cardiocrinum* resources and habitats have suffered considerable damage [7]. For example, local residents collect *Cardiocrinum* bulbs to make starch for consumption and sale. In some scenic spots, the inflorescences of common *Cardiocrinum* are broken by humans, and some *Cardiocrinum* or their bulbs are eaten by animals. This has resulted in a dramatic decline in both the population size and density of wild *Cardiocrinum*, with most remaining individuals existing as isolated solitary plants or in small clusters of fewer than a dozen [1]. Therefore, fully understanding the distribution of *Cardiocrinum* and the ecological environment suitable for their growth will be beneficial to the protection of the diversity of wild *Cardiocrinum.* This will also allow the development and utilization of their medicinal resources, the domestication and artificial cultivation of *Cardiocrinum*, the introduction of new species, and the protection of plant resources.

Climate serves as the primary determinant governing species distribution patterns and ecosystem processes [8]. Climate change-induced alterations in temperature and precipitation regimes significantly modify plant physiological processes, thereby influencing growth, developmental patterns, reproductive success, population stability, and geographic distribution ranges [9,10]. Plants may respond to climatic changes through either phenotypic plasticity and local adaptation or by altering their geographic distributions through range shifts and contractions [11,12]. Thus, >80% of plant species may face the alterations in diversity, current distribution, and potential habitats [13]. For example, *Forsythia suspensa* has shown a northward shift in its potential habitat in China [14], and *Haloxylon* in Central Asia may move to higher altitudes under a warming climate [15].

Species distribution models (SDMs) are grounded in niche theory, utilizing known occurrence records and associated environmental variables to identify key distribution drivers and predict potential species ranges [16,17]. SDMs mainly include BIOCLIM [18], Ecological Niche Factor Analysis (ENFA) [19], the Generalized Additive Model (GAM) [20], the Generalized Linear Model (GLM) [21], Maximum Entropy (MaxEnt) [22], Classification and Regression Tree (CART) [23], etc. Among these approaches, the Maximum Entropy (MaxEnt) model, grounded in the principle of maximum entropy, represents one of the most widely used and robust methods for modeling species’ geographical distributions [24]. The MaxEnt algorithm utilizes species occurrence records and environmental covariates to estimate a probability distribution with maximum entropy, enabling the analysis and prediction of potential species distribution patterns [25]. Among these models, the MaxEnt model can better combine the distribution of species with environmental and performs well even in the absence of species distribution [26]. It offers computational efficiency and user-friendly implementation, making it particularly suitable for modeling both realized and potential species distributions [27].

To assess the habitat distribution patterns and key environmental determinants of habitat suitability for *Cardiocrinum* in China, we employed the MaxEnt modeling approach to predict their potential geographic distributions. Therefore, the present study aimed to achieve the following objectives: (1) to determine environmental factors impacting distribution of *Cardiocrinum* species; (2) to predict suitable habitats for *Cardiocrinum* species under diverse scenarios by using current simulation and climate change; (3) to determine the distribution of *Cardiocrinum* species under future climatic conditions to provide an effective basis for its effective conservation, introduction, and utilization.

## 2. Materials and Methods

### 2.1. The Source and Acquisition of Cardiocrinum Data

**Figure 1:**
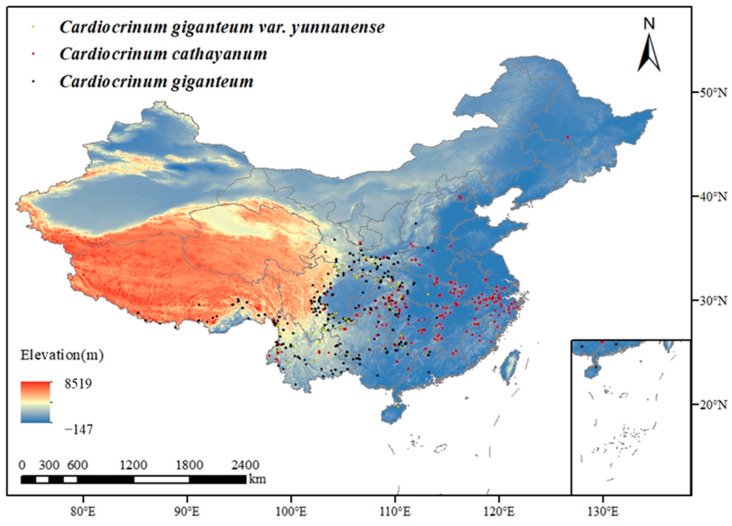
Distribution map of *Cardiocrinum* in China.

The distribution information of *Cardiocrinum* was obtained by consulting the Global Biodiversity Information Facility (GBIF) (https://www.gbif.org/, accessed on 21 March 2024), the Digital Herbarium of China (https://www.cvh.ac.cn/?from=singlemessage, accessed on 21 March 2024), and the photo distribution map of Flora of China (https://www.iplant.cn/, accessed on 21 March 2024). Finally, 888 effective distribution points of *C. cathayanum*, 1392 effective distribution points of *C. giganteum*, and 126 effective distribution points of *C. giganteum* var. *yunnanense* in China were obtained (Figure 1). The selected distribution point data were then made into a csv file with species names and the distribution of longitude and latitude points.

### 2.2. Environmental Parameters

Current and future climate data were obtained from WorldClim data website (https://www.worldclim.org/data/index.html, accessed on 12 April 2024), including 19 environmental variables (bio1-bio19) with a 30 s (ca.1 km) spatial resolution and three terrain variables: elevation (elev), aspect (asp), and slope (slo). These were collected using Computer Network Informations Center of the Chinese Academy of Sciences and the International Scientific Data Website (https://www.gscloud.cn/, accessed on 12 April 2024). As well as this, human activity (ha) data, from the Global Human Impact Index, were from the Socioeconomic Data and Applications Center (http://sedac.ciesin.columbia.edu/wildareas/, accessed on 12 April 2024) (Table 1).

The climate scenarios proposed by CMIP6 based on different socioeconomic development and anthropogenic emission pathways are called “Shared Socioeconomic Pathways” (SSPs). According to the intensity of emissions, three shared socioeconomic pathways were selected, including SSP126 (lowest greenhouse gas emission scenario), SSP245 (moderate greenhouse gas emission scenario), and SSP585 (highest greenhouse gas emission scenario). For future climate modeling, we chose the BCC-CSM2-MR data set to project scenarios for the 2040s (2041–2060) and 2080s (2081–2100). ArcGIS 10.8 was used for the extraction of environmental variables based on the boundaries of the administrative division map of China, and then they were converted into ASCII format for backup.

### 2.3. Maximum Entropy Model (MaxEnt) Simulation

The predictive performance of the mode was determined by using the Area Under the Receiver Operating Characteristic Curve (AUC). The value range from 0.5 to 1 shows random prediction and perfect discrimination, respectively [28]. The Area Under the Curve (AUC) values ranged from 0.5 (random prediction) to 1.0 (perfect discrimination) [29]. Higher AUC values correspond to greater predictive reliability [30,31]. Following established ecological modeling standards, we classified prediction accuracy into five categories: excellent (0.9–1.0), good (0.8–0.9), fair (0.7–0.8), poor (0.6–0.7), and fail (0.5–0.6) [32]. The MaxEnt model outputs in logistic format were geoprocessed using ArcGIS 10.8. Using China’s administrative boundary layer, we extracted and classified the potential suitable habitats for *Cardiocrinum*, which were subsequently visualized as a spatial distribution map.

The SDM tool in ArcGIS 10.8 software was used to quantify the suitable habitat of *Cardiocrinum* based on the results of the MaxEnt model and set the value that excluded 10% of the position with the lowest predicted value as the threshold. Therefore, the potential suitable habitat of this species was divided into three different levels. The first level was non-suitable with a <10 percentile training presence, the second was suitable habitat with a <10 percentile training presence–0.66 and third level was highly suitable habitat with a percentile presence > 0.66. Thereafter, the suitable habitat area for *Cardiocrinum* was calculated. The ASCII result file output from the MaxEnt model was extracted using ArcGIS 10.8 software. The spatial analysis and visualization mapping were performed using the reclassification tool to calculate the area changes in different levels of suitable habitats of *Cardiocrinum* under current climate scenarios and future times. Among them, −1 represented a newly added suitable habitat, 0 represented a non-suitable habitat, 1 represented a retained suitable habitat, and 2 represented a lost suitable habitat. The data were converted into binary data using the threshold (suitable habitat and non-suitable habitat), and the “Centroid Changes (Lines)” analysis tool in the SDM Toolbox v2.6 of ArcGIS 10.8 software was used to calculate the location of the distribution center of the suitable habitats and the direction of its spatial migration change.

The niche consistency test among *Cardiocrinum* was performed using ENMTools [33]. In ENMTools 1.1.2 software, MaxEnt was used to generate a layer under current climate conditions to calculate the actual values of Schoener D [34] and Warren I [35], and the distribution frequency of the expected values of Schoener D and Warren I was obtained by running the pseudo-replication data set 100 times. The statistical significance between observed and expected values of the evaluation index was assessed using a non-parametric Monte Carlo permutation test. When the actual I and D values were significantly lower than the expected values of the pseudo-replication data set (*p* < 0.01), the hypothesis of niche consistency was rejected, indicating that niche differentiation occurred between the two [36,37].

## 3. Results

### 3.1. Model Accuracy Assessment

The MaxEnt model demonstrated exceptional predictive performance, with training set AUC values exceeding 0.98 for all species (Table 2). These results indicate the model achieved excellent discrimination capacity (AUC > 0.9 is considered outstanding) and accurately simulated *Cardiocrinum*’s potential habitat distribution patterns.

### 3.2. Key Environmental Variables

**Figure 2:**
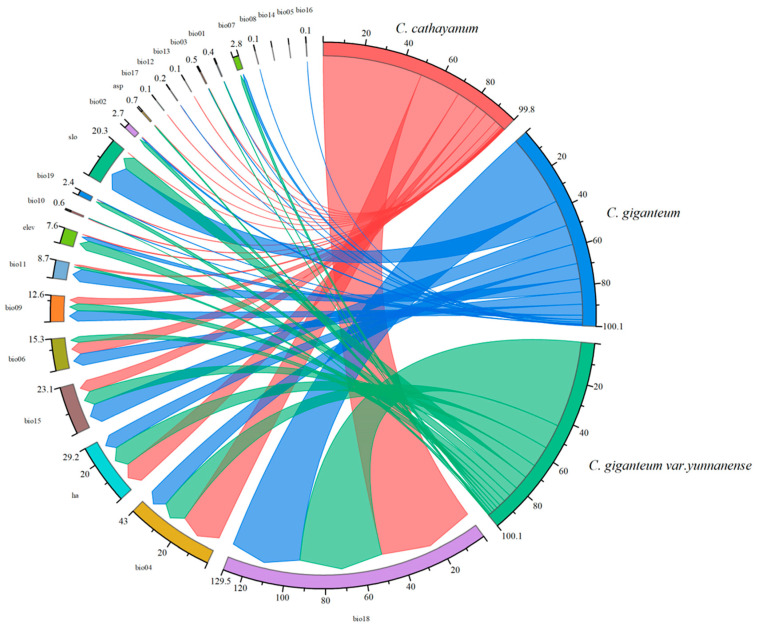
The percentage contributions of environmental factors for *Cardiocrinum*.

The MaxEnt model analysis identified five key environmental variables that significantly affect the distribution habitat of *Cardiocrinum.* Among the 23 variables examined were the following: warmest-quarter precipitation (bio18), temperature seasonality (bio04), the human activity index (ha), precipitation seasonality (bio15), and slope (slo) (in order of relative contribution). Among these environmental determinants, precipitation emerged as the predominant factor governing *Cardiocrinum* distribution patterns. (Figure 2). The Jackknife test results showed that for *C. cathayanum*, the sum of the contribution rates of warmer-quarter rainfall (bio18) and temperature seasonality (bio4) reached 70.8%; the warmest-season precipitation was 446.3–680.8 mm, and the temperature seasonality range was 738.5–912.8. For the *C. giganteum*, the sum of the precipitation of the warmest quarter (bio18), slope (slo), and precipitation seasonality (bio15) contributed 59.1%; the optimum range of precipitation was 431.6–1995.3 mm, the slope range was 5.45–5.53, and the seasonal variation range of precipitation was 61.69–94.30. The combined contribution of warmest-quarter rainfall and seasonal temperature (bio18) accounted for 56.2% of the observed distribution patterns in *C. giganteum* var. *yunnanense*; the optimum range of precipitation was 451.2–754.1 mm, and the seasonal variation range of temperature was 485.48–839.74.

### 3.3. The Distribution of the Suitable Area of Cardiocrinum Under the Current Climate

**Figure 3:**
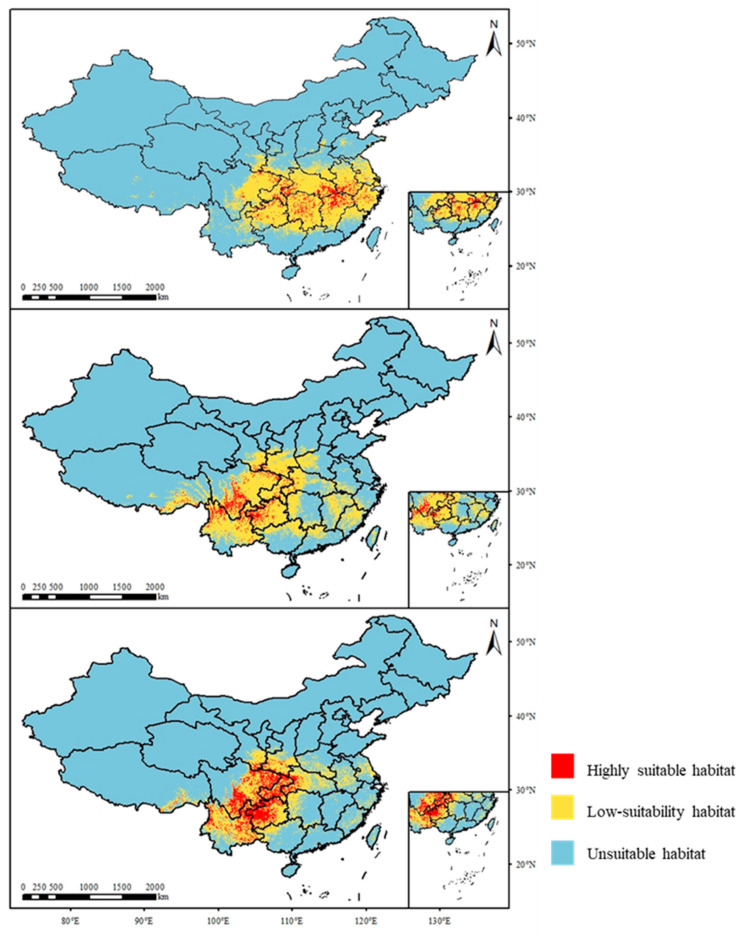
The distribution of *Cardiocrinum* under the current climate.

The distribution of three species of *Cardiocrinum* in China at present is shown in Figure 3. *C. cathayanum* is widely present in central and eastern China, and its most suitable areas are in Hubei, northern Jiangxi, southern Anhui, and central Hunan. The total suitable area is 155.32 × 10^4^ km^2^; *C. giganteum* is present in the southwest and east parts of northwest areas and sporadically distributed in central China and east China, with its highly suitable areas mainly in southern Qinghai, northwest and northeast Yunnan, and western Guizhou, with a total suitable area of 165.06 × 10^4^ km^2^; *C. giganteum* var. *yunnanense* is mainly distributed in the southwest, with its highly suitable areas concentrated in Chongqing, Guizhou, and southeastern Qinghai, with a total suitable area of 133.74 × 10^4^ km^2^.

### 3.4. Potential Habitat Change for Cardiocrinum in the Future

Compared with the current period, the suitable area of *C. cathayanum* increased under the 2041–2060 SSP126 climate scenario and then decreased under the 2081–2100 SSP126 scenario. The highly suitable habitat along the Yangtze River Basin first increased under the 2041–2060 SSP126 climate scenario and then decreased under the 2081–2100 SSP126 climate scenario. The total suitable habitat area increased to 206.89 × 10^4^ km^2^ and then decreased to 196.48 × 10^4^ km^2^. The highly suitable habitat area increased to 35.66 × 10^4^ km^2^ and then decreased to 14.74 × 10^4^ km^2^. The total suitable habitat area of *C. giganteum* depicted an increasing trend, and its total suitable area increased to 191.30 × 10^4^ km^2^. Further, the total suitable area of *C. giganteum* var. *yunnanense* first declined under the 2041–2060 SSP126 climate scenario. Then, it substantially increased under the 2081–2100 SSP126 climate scenario and reached 138.43 × 10^4^ km^2^. In general, compared with the current situation, the suitable habitat areas for *Cardiocrinum* show an increasing trend.

Under the 2041–2060 to 2081–2100 SSP245 climate scenario, the suitable habitat areas of *C. cathayanum* and *C. giganteum* increased overall, while the suitable habitat area of *C. giganteum* var. *yunnanense* decreased. The total suitable habitat areas of *C. cathayanum* and *C. giganteum* increased to 217.53 × 10^4^ km^2^ and 227.32 × 10^4^ km^2^, respectively; under the 2041–2060 SSP126 climate scenario, the total suitable habitat area of *C. giganteum* var. *yunnanense* increased to 140.14 × 10^4^ km^2^, while under the 2081–2100 SSP126 climate scenario, it decreased to 128.65 × 10^4^ km^2^.

**Figure 4:**
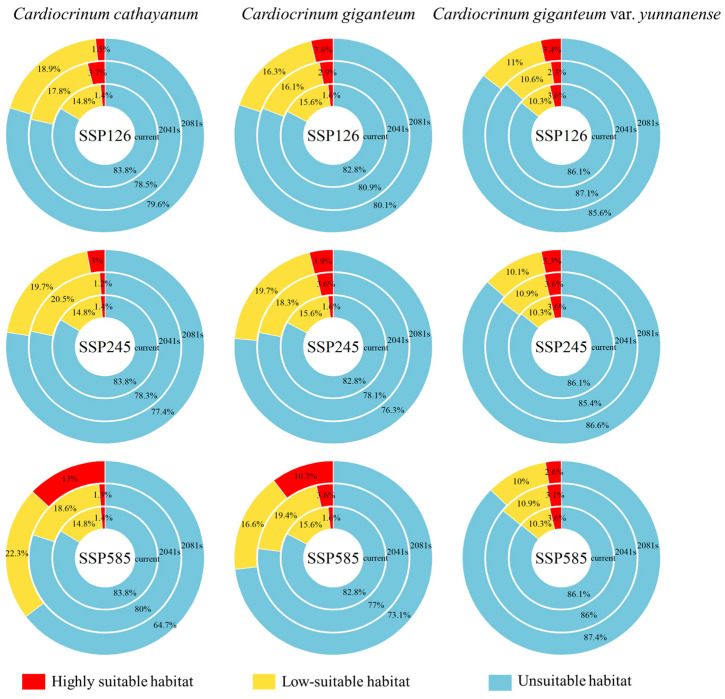
The change in the suitable habitat area of *Cardiocrinum* under future climatic conditions.

Under the 2041–2060 to 2081–2100 SSP585 climate scenario, in general, the suitable habitat area of *C. cathayanum* and *C. giganteum* giant increased significantly, while that of *C. giganteum* var. *yunnanense* decreased slightly. The area suitable for the *C. cathayanum* habitat reached 339.29 × 10^4^ km^2^. The total suitable habitat of *C. giganteum* increased to 258.57 × 10^4^ km^2^, and the highly suitable habitat was 98.84 × 10^4^ km^2^. The total suitable habitat of *C. giganteum* var. *yunnanense* finally decreased to 121.38 × 10^4^ km (Figure 4).

### 3.5. Migration of Centroid of Cardiocrinum-Suitable Area Under Different Climate Scenarios

**Figure 5:**
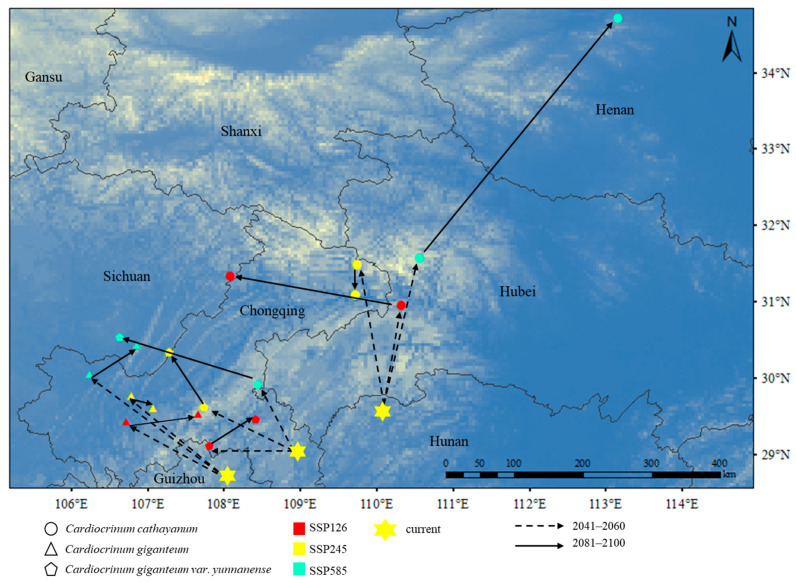
Trajectory changes in the centroid in the future for *Cardiocrinum*.

The current center of mass of *C. cathayanum* is located in Sangzhi County, Zhangjiajie City, Hunan Province (110°03′46.6596″ E, 29°35′44.9952″ N). The center of mass of *C. giganteum* is located in Wuchuan County, Zunyi City, Guizhou Province (108°04′44″ E, 28°42′11″ N). The center of mass of *C. giganteum* var. *yunnanense* is located in Youyang County (108°57′58.6800″ E, 29°04′26″ N). In the SSP126 and SSP245 scenarios, the centroid of *C. cathayanum* shifted to the northwest side, while in SSP585 scenario, the centroid shifted to the northeast. In these scenarios, the center of mass of *C. giganteum* and *C. giganteum* var. *yunnanense* shifted to the northwest (Figure 5).

### 3.6. Ecological Niche Differentiation

**Figure 6:**
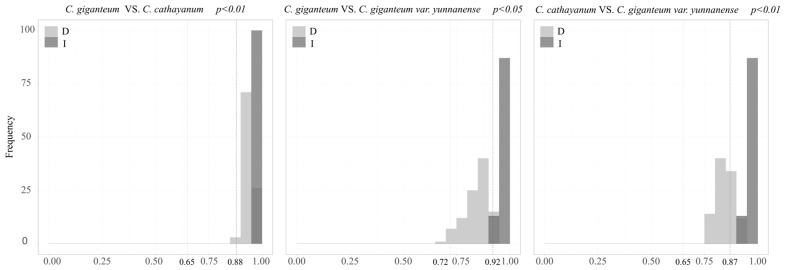
Niche identity test for *Cardiocrinum*. Note: The vertical dotted lines show the empirical values of Schoener D and Warren I, and the histograms represent the frequency of the expected Schoener D and Warren I.

The niche consistency test showed results for *C. giganteum* and *C. cathayanum* (D: 0.65; I: 0.88), *C. giganteum* and *C. giganteum* var. *yunnanense* (D: 0.72; I: 0.92), and *C. cathayanum* and *C. giganteum* var. *yunnanense* (D: 0.65; I: 0.87). The actual values of Warren I and Schoener D for *C. cathayanum* and the other two were lower than the expected values, indicating that there was a significant niche change between *C. cathayanum* and the others. There was an overlapping area between *C. giganteum* and *C. giganteum* var. *yunnanense* in Warren I and Schoener D, indicating that they had overlapping niches (Figure 6).

## 4. Discussion

Temperature and rainfall are the most impact factors regulating plant growth and development [38]. *Cardiocrinum* are typical understory herbaceous plants, and the growth of understory herbaceous plants is largely affected by environmental factors and stand factors [39]. Environmental factors affect forest stand factors in the environment by affecting the height and density of trees, namely water and heat factors, soil nutrients, and understory light, which, in turn, affect understory herbaceous plants [40]. Light availability, soil nutrient content, and water supply emerge as the principal abiotic factors governing understory plant distribution patterns and diversity dynamics [41,42,43]. Previous studies have shown that *Cardiocrinum* mostly grow on natural forest slopes below 1000 m above sea level, while *C. giganteum* grows on natural forest steep slopes at 1300–1600 m above sea level [44]. There are usually mountain streams and creeks near their wild habitats, and the air humidity is very high. Judging from the habitat of wild *Cardiocrinum*, they can tolerate a local extreme minimum temperature of −10–6 °C, and the soil requires good drainage and is not resistant to strong direct sunlight [2]. Therefore, *Cardiocrinum* need appropriate moisture for growth. Appropriate precipitation can promote the growth and reproduction of *Cardiocrinum*. Insufficient precipitation may lead to the insufficient development of their bulbs, while excessive precipitation may cause bulb rot. Previous studies indicate that *Cardiocrinum* species thrive in regions with annual rainfall of 600–2200 nm and the annual average relative humidity is above 70%. High precipitation is not conducive to the development of fruits, while higher annual average temperatures are more conducive to the development of fruits and seeds [1]. The sensitivity to temperature may be due to the morphological physiological dormancy (MPD) of their seeds [45]. After the seeds of *Cardiocrinum* mature, the embryo is not fully developed, so a complex temperature stratification from high temperature to low temperature is required to effectively break the dormancy of *Cardiocrinum* seeds [46]. Therefore, temperature changes are of great significance to the growth of *Cardiocrinum*.

The future distribution of the *C. giganteum* and *C. giganteum* var. *yunnanense* will also be affected by slope and human activity factors. The influence of slope may be related to the fact that these two *Cardiocrinum* are distributed in high-altitude areas. The slope will affect the light of the plants, the water content and saturation of the land, and excessive precipitation will form runoff, leading to soil erosion [47]. Therefore, the decrease in soil moisture and fertility will affect the growth and reproduction of them, thereby affecting their distribution.

*Cardiocrinum* serve multiple purposes as ornamental plants for garden landscaping and pot cultivation [5]. Future expansion and northward shifts in the suitable habitat range of *C. cathayanum* may be influenced by human cultivation. Additionally, these plants have medicinal and edible applications [48]. *C. giganteum* and *C. giganteum* var. *yunnanense* are primarily found in the Yunnan–Guizhou–Sichuan region, where their bulbs have traditionally been consumed. This practice, along with economic development, agricultural expansion, and habitat destruction, threatens wild populations by depleting local resources and degrading their natural habitats.

*C. cathayanum* exhibits a primary distribution in eastern and central China, with core populations concentrated in the provinces of Hubei, Hunan, Jiangxi, Zhejiang, Anhui, and Jiangsu [49]. *C. giganteum* is primarily distributed across southwestern to central China, with its core range encompassing Yunnan, Tibet, Sichuan, Shaanxi, Hubei, and Henan Provinces. Sichuan is the place with the most densely distributed *C. giganteum* in China [50]. *C. giganteum* var. *yunnanense* is predominantly distributed in the southwestern regions of China, with its core range encompassing Sichuan and Yunnan Provinces. Model fitting results indicate the medium- and high-suitability habitat area of *C. cathayanum* is about 155.32 × 10^4^ km^2^, the medium- and high-suitability habitat area of *C. giganteum* is about 165.06 × 10^4^ km^2^, and the medium- and high-suitability habitat area of *C. giganteum* var. *yunnanense* is about 133.74 × 10^4^ km^2^. The suitable area is basically consistent with the model fitting, and the model fitting degree is high. Under the SSP126, SSP245, and SSP585 climate scenarios, projections for 2041–2060 and 2081–2100 indicate that the models project an expansion of suitable habitats for both *C. cathayanum* and *C. giganteum* across all climate scenarios, with *C. cathayanum* demonstrating particularly significant gains. The results show that the suitable area for *C. giganteum* increased in SSP126 climate scenario, while it decreased in SSP245 and SSP585 scenarios. This decrease in suitable area might be linked with its narrow ecological adaptability. Narrowly distributed plant species typically exhibit limited ecological plasticity, rendering them more vulnerable to climate change impacts compared to widely distributed taxa [51].

From the perspective of the future changes in the centroid migration, the centroids of *Cardiocrinum* all showed a trend of migrating to higher latitudes. The centroids of *C. giganteum* and *C. giganteum* var. *yunnanense* had a smaller range of movement, and the upward migration did not exceed 2 degrees of latitude, while the centroid of *C. cathayanum* leaves had a significant northward migration under the 2081s SSP585 scenario, exceeding 5 degrees of latitude. The reason for this difference may be that *C. cathayanum* is more affected by temperature and precipitation, and plants that are more sensitive to temperature will migrate more significantly to the north [52]. In addition to these two factors, *C. giganteum* and *C. giganteum* var. *yunnanense* are also affected by many other factors. In addition, the centroid longitude of the *C. giganteum* and *C. giganteum* var. *yunnanense* moved westward in all three climate scenarios. This may be because the longitude of alpine plants moves westward when the distribution of alpine plants moves upward in altitude [53]. It is speculated that due to climate warming, plants will migrate to higher altitudes and higher latitudes [54,55,56]. In the future climate warming, northern China will become humid and warm [57]. The projected increase in warm, humid areas is expected to significantly expand suitable habitats for *Cardiocrinum*. Model simulations indicate that these favorable conditions will facilitate both northeastward and altitudinal range shifts in the species’ distribution. In addition, under the condition of global warming, China‘s forest vegetation will tend to move north [58]. As understory plants, *Cardiocrinum* may move northward with vegetation in order to survive better. This observed migration pattern aligns with well-documented biogeographic responses to climate change, where species increasingly shift toward higher latitudes and elevations under warming conditions [59].

Interspecific niche differentiation primarily arises from divergence in both fundamental niches and species-specific environmental contexts [35]. There is no overlapping niche between *C. cathayanum* and the other two species. This may be due to the differences in influencing factors and suitable habitats between *C. cathayanum* and the others. *C. cathayanum* is primarily not affected by slope, while *C. cathayanum* and *C. giganteum* var. *yunnanense* are greatly affected by slope. The highest habitat suitability for *C. cathayanum* occurs predominantly in lowland floodplains of the middle and lower Yangtze River Basin, while the high-suitability areas of *C. giganteum* and *C. giganteum* var. *yunnanense* are around the Sichuan Basin. Studies have shown that the interaction between geological changes in the eastern part of the Qinghai–Tibet Plateau and climate change in the late Miocene induced differences among *C. cathayanum* and *C. giganteum* [60,61]. Therefore, environmental heterogeneity may cause significantly different microenvironments in different populations, which, in turn, leads to differences in local genetic variation and differentiation in local environmental adaptability [62]. This shows that environmental factors play a crucial role in species differentiation. *C. giganteum* and *C. giganteum* var. *yunnanense* have overlapping ecological niches, which may be because *C. giganteum* var. *yunnanense* is a variant of *C. giganteum*. The distribution of the area of both these species overlaps because of the same environmental factors. This overlap may increase the competitive pressure between the two species, especially when resources are limited or the environment is volatile. However, niche overlap does not necessarily lead to direct competitive exclusion, because species can also reduce direct competition and achieve coexistence through niche differentiation [63].

## 5. Conclusions

Our findings indicate that current *C. cathayanum* distribution is primarily concentrated in the middle and lower Yangtze River Basin, while *C. giganteum* and *C. giganteum* var. *yunnanense* are distributed radially along the Sichuan Basin. The distributions of *C. cathayanum* and *C. giganteum* are predominantly constrained by seasonal temperature and the rainfall of the warmest quarter (bio18). In addition, the suitable habitats of *C. giganteum* and *C. giganteum* var. *yunnanense* are also affected by slope and human activity. Under future climate scenarios (SSP126, SSP245, and SSP585) for both the 2041–2060 and 2081–2100 periods, the suitable habitat areas of *C. giganteum* and *C. giganteum* will gradually increase, while that of *C. giganteum* var. *yunnanense* will decrease. *C. cathayanum* will have a tendency to migrate northward, while the distribution of *C. giganteum* will radiate along the current suitable habitat. There is niche differentiation between *C. cathayanum* and *C. giganteum* and *C. giganteum* var. *yunnanense*, while there is no niche differentiation between *C. giganteum* and *C. giganteum* var. *yunnanense*. This may be caused by differences in the environment. In order to better protect the resources of *Cardiocrinum* in China, we must first avoid uncontrolled excavation. Secondly, we can establish corresponding seed and seedling breeding bases in their suitable habitats and actively carry out the investigation, preservation, and collection of wild germ-plasm resources. At the same time, we can also promote research on the reproductive model of *Cardiocrinum* growth in its highly suitable habitats through various means such as tissue culture, to provide guarantees for the sustainable use and protection of *Cardiocrinum*.

## Figures and Tables

**Table 1:** The 23 environmental variables used for model prediction.

Variables	Abbreviations
Annual mean temperature	bio1
Mean diurnal range	bio2
Isothermality	bio3
Temperature seasonality	bio4
Max. temperature of warmest month	bio5
Min. temperature of coldest month	bio6
Temperature annual range	bio7
Mean temperature of wettest quarter	bio8
Mean temperature of driest quarter	bio9
Mean temperature of warmest quarter	bio10
Mean temperature of coldest quarter	bio11
Annual precipitation	bio12
Precipitation of wettest month	bio13
Precipitation of driest month	bio14
Precipitation seasonality	bio15
Precipitation of wettest quarter	bio16
Precipitation of driest quarter	bio17
Precipitation of warmest quarter	bio18
Precipitation of coldest quarter	bio19
Elevation	elev
Human activity	ha
Slope	slo
Aspect	asp

**Table 2:** AUC and TSS value for *Cardiocrinum*.

Species	AUC Training	AUC Test	TSS
*C. cathayanum*	0.989	0.983	0.913
*C. giganteum*	0.982	0.980	0.936
*C. giganteum* var. *yunnanense*	0.995	0.996	0.989

## Data Availability

Data can be made available on reasonable request.
